# Genetic knockout of myosin light chain kinase (MLCK210) prevents cerebral microhemorrhages and attenuates neuroinflammation in a mouse model of vascular cognitive impairment and dementia

**DOI:** 10.1007/s11357-019-00072-4

**Published:** 2019-05-19

**Authors:** David J. Braun, Adam D. Bachstetter, Tiffany L. Sudduth, Donna M. Wilcock, D. Martin Watterson, Linda J. Van Eldik

**Affiliations:** 1 Sanders-Brown Center on Aging, University of Kentucky, 101 Sanders-Brown Bldg., 800 S. Limestone Street, Lexington, KY 40536 USA; 2 Department of Neuroscience, University of Kentucky, Lexington, KY 40536 USA; 3 Spinal Cord and Brain Injury Research Center, University of Kentucky, Lexington, KY 40536 USA; 4 Department of Physiology, University of Kentucky, Lexington, KY 40536 USA; 5 Department of Pharmacology, Northwestern University Feinberg School of Medicine, Chicago, IL 60611 USA

**Keywords:** Myosin light chain kinase, Cerebrovascular, Microhemorrhage, Neuroinflammation, Vascular cognitive impairment and dementia, Knockout

## Abstract

The blood-brain barrier (BBB) is critical in maintenance of brain homeostasis, and loss of its functional integrity is a key feature across a broad range of neurological insults. This includes both acute injuries such as traumatic brain injury and stroke, as well as more chronic pathologies associated with aging, such as vascular cognitive impairment and dementia (VCID). A specific form of myosin light chain kinase (MLCK210) is a major regulator of barrier integrity in general, including the BBB. Studies have demonstrated the potential of MLCK210 as a therapeutic target for peripheral disorders involving tissue barrier dysfunction, but less is known about its potential as a target for chronic neurologic disorders. We report here that genetic knockout (KO) of MLCK210 protects against cerebral microhemorrhages and neuroinflammation induced by chronic dietary hyperhomocysteinemia. Overall, the results are consistent with an accumulating body of evidence supporting MLCK210 as a potential therapeutic target for tissue barrier dysfunction and specifically implicate it in BBB dysfunction and neuroinflammation in a model of VCID.

## Introduction

Loss of blood-brain barrier (BBB) functional integrity is a major pathological component of age and disease-associated dementia (Zlokovic 2008; Daneman 2012), and the severity of barrier dysfunction is often associated with worsened cognitive function (Montagne et al. 2015; Janelidze et al. 2017). Attenuation or rescue of BBB dysfunction is therefore a promising target for treatment of a variety of neurodegenerative diseases disproportionately affecting older adults. The BBB is a specialized system of endothelial cell junctions and glial associations that are dynamically regulated by a signaling network composed of discrete regulatory points (for review see Daneman and Prat 2015). For example, myosin light chain kinase (MLCK) is a signaling protein involved in regulation of barrier integrity that has been identified as a potential therapeutic target in disease-associated tissue barrier dysfunction. There are multiple MLCK proteins, but the focus of the present study is a 210-kDa MLCK protein encoded by the *mylk1* genetic locus, referred to as MLCK210 (for review see Khapchaev and Shirinsky 2016). Prior studies on the role of MLCK210 in tissue barrier dysfunction and the potential of selective inhibitors have largely focused on non-CNS disorders (for recent reviews see Cunningham and Turner 2012; Rigor et al. 2013; Khapchaev and Shirinsky 2016; Xiong et al. 2017), including acute lung injury models (Wainwright et al. 2003; Rossi et al. 2007; Mirzapoiazova et al. 2011; Usatyuk et al. 2012; Fazal et al. 2013; Wang et al. 2014; Wang et al. 2016; Zhou et al. 2015), burn injury (Reynoso et al. 2007; Guo et al. 2012; Zahs et al. 2012), acute diarrhea (Clayburgh et al. 2005; Clayburgh et al. 2006), endotoxic shock (Ralay Ranaivo et al. 2007; Gaceb et al. 2016), cardiovascular shear stress (Ohlmann et al. 2005), atherosclerosis (Sun et al. 2011), hypoxia (Arnaud et al. 2018), and intestinal injury models (Al-Sadi et al. 2012; Gilbert et al. 2012; Wu et al. 2014; Lorentz et al. 2017; Nighot et al. 2017; Al-Sadi et al. 2019). Additionally, there exists a smaller literature exploring the benefit of inhibition of MLCK in the context of BBB dysfunction. This includes in vivo models of traumatic brain injury (Luh et al. 2010; Rossi et al. 2013), cerebral ischemia (Zhang et al. 2015), subarachnoid hemorrhage (Luh et al. 2018), and in vitro experiments modeling cerebral hypoxia (Kuhlmann et al. 2007; Hicks et al. 2010) and cytokine elevation (Huppert et al. 2010; Beard et al. 2014). The in vivo data show MLCK suppression can ameliorate acute cerebrovascular injury, while the in vitro data suggest a link to chronic stressors commonly underlying cerebrovascular dysfunction. To extend these findings, we performed comparative studies of the MLCK210 KO mouse response to a diet-induced hyperhomocysteinemia (HHcy) model of chronic VCID. This model uses a B vitamin-deficient diet to induce elevated levels of plasma homocysteine, which leads to progressive BBB dysfunction and reproducible and quantitative cerebrovascular changes that mimic many of those found in clinical VCID (for review see Price et al. 2018). Thus, subjecting the MLCK210 KO model to the diet-induced HHcy model of chronic VCID allows a direct test of the hypothesis that MLCK210 is a viable target for progressive CNS diseases such as VCID. We report here that MLCK210 KO mice are protected from HHcy-induced microhemorrhage formation and pro-inflammatory biomarker changes, justifying further exploration of MLCK210 inhibition as a therapeutic strategy for chronic neurological diseases involving a BBB dysfunction mechanism.

## Methods

### Animals and experimental diet

The experiment was carried out in a 2 × 2 diet by genotype design. All animals received 6 weeks of the HHcy diet (Envigo, #TD.97345)—deficient in vitamins B_6_, B_9_, and B_12_ with excess methionine—or nutritionally matched control diet with normal methionine and vitamin levels (Envigo, #TD.01636) (Sudduth et al. 2013; Sudduth et al. 2014; Sudduth et al. 2017). C57BL/6J mice (The Jackson Laboratory strain #664) were used as wild-type (WT) controls for the MLCK210 KO mice that were generated as previously reported (Wainwright et al. 2003). Eight MLCK210 KO mice (4 male/4 female) received control diet, and 8 (4M/4F) received HHcy diet. Eight WT mice (3M/5F) received control diet, and 10 (3M/7F) received HHcy diet. Animals were housed 1–4 per cage (503.22 usable cm^2^) in a room at 23 °C ± 2 °C, under a 14/10-h light/dark cycle beginning at 6:00 AM. All mice were administered experimental diet between 2 and 3 months of age and were sacrificed at 3.5–4.5 months of age after 6 weeks on diet. Mice had ad libitum access to water and chow.

### Tissue collection

Mice were deeply anesthetized with 5% isoflurane and arterial blood collected from the left ventricle for measurement of homocysteine levels by the University of Kentucky Hospital clinical laboratory. Mice subsequently underwent transcardial perfusion with 50 ml ice-cold phosphate-buffered saline (PBS) at a flow rate of 10 ml/min before decapitation and brain removal and dissection. The left hemisphere was post-fixed in 4% paraformaldehyde for 24 h at 4 °C and cryo-protected in 30% sucrose for 48 h at 4 °C before sectioning. A portion of frontal cortex from the right hemisphere was dissected, flash frozen in liquid nitrogen, and stored at − 80 °C until processing for biochemistry.

### Immunohistochemistry for Prussian blue

The left hemisphere was cut coronally into 30 μm sections, with every 24th section collected for staining. A total of 5–7 sections per hemibrain were mounted and stained for hemosiderin using Prussian blue as described previously (Wilcock et al. 2004). Slides were incubated in a 2% potassium ferrocyanide in 2% hydrochloric acid solution for 15 min, followed by a counterstain in a 1% neutral red solution for 10 min. The number of Prussian blue positive profiles were counted across each section, and an average per-section value was generated for analysis.

### Quantitative reverse-transcriptase polymerase chain reaction

Quantitative reverse-transcriptase polymerase chain reaction (qRT-PCR) was performed as previously reported (Sudduth et al. 2013), with all reagents acquired from Thermo Scientific (Rockford, IL, USA). Briefly, RNA was extracted from the right frontal cortex using the Trizol plus RNA purification system according to the manufacturer’s instructions. Total RNA was quantified with a nanodrop spectrophotometer, and cDNA made using the cDNA High Capacity kit according to instructions. Real-time PCR was performed using the TaqMan Gene Expression assay kit, and genes normalized to 18s rRNA. TaqMan probes were used to measure transcript levels of Arg1 (Mm00475988_m1), IL-1β (Mm00434228_m1), IL-10 (Mm00439616_m1), IL-12A (Mm00434165_m1), IL-1Ra (Mm00446186_m1), IL-6 (Mm00446190_m1), MMP2 (Mm00439506_m1), MMP3 (Mm00440295_m1), MMP9 (Mm00600163_m1), MMP14 (Mm00485054_m1), TIMP1 (Mm00441818_m1), TIMP2 (Mm00441825_m1), TNF-α (Mm00443258_m1), and YM1 (Mm00657889_mH). Fold change values were determined for mice receiving experimental diet relative to mice receiving control diet within the same genotype. For comparisons between WT and MLCK210 KO mice on control diet, fold change values were determined for the KO mice relative to WT. All fold change values were calculated using the 2^(−ΔΔCt)^ method, and log2 normalized.

### Statistical analyses

Statistical analyses and figure generation were performed using GraphPad Prism 7 (GraphPad Software, La Jolla, CA, USA). Two-way analysis of variance (ANOVA) with Sidak’s post hoc testing was performed for most comparisons, with a statistical significance level set to *α* = .05. For comparison of gene expression between WT and MLCK210 KO mice on control diet, *t* tests were performed for each gene, followed by the two-stage step-up method (Benjamini et al. 2006) to control false discovery rate, with *Q* = 10%. All graphs show means with error bars representing the standard error of the mean (SEM). Where reported in the text, data are described with M = mean, SD = standard deviation, and CI = 95% confidence interval.

## Results

### Experimental diet induces HHcy in both MLCK210 KO and WT mice, but MLCK210 KO mice are protected from HHcy-induced microhemorrhages

**Fig. 1:**
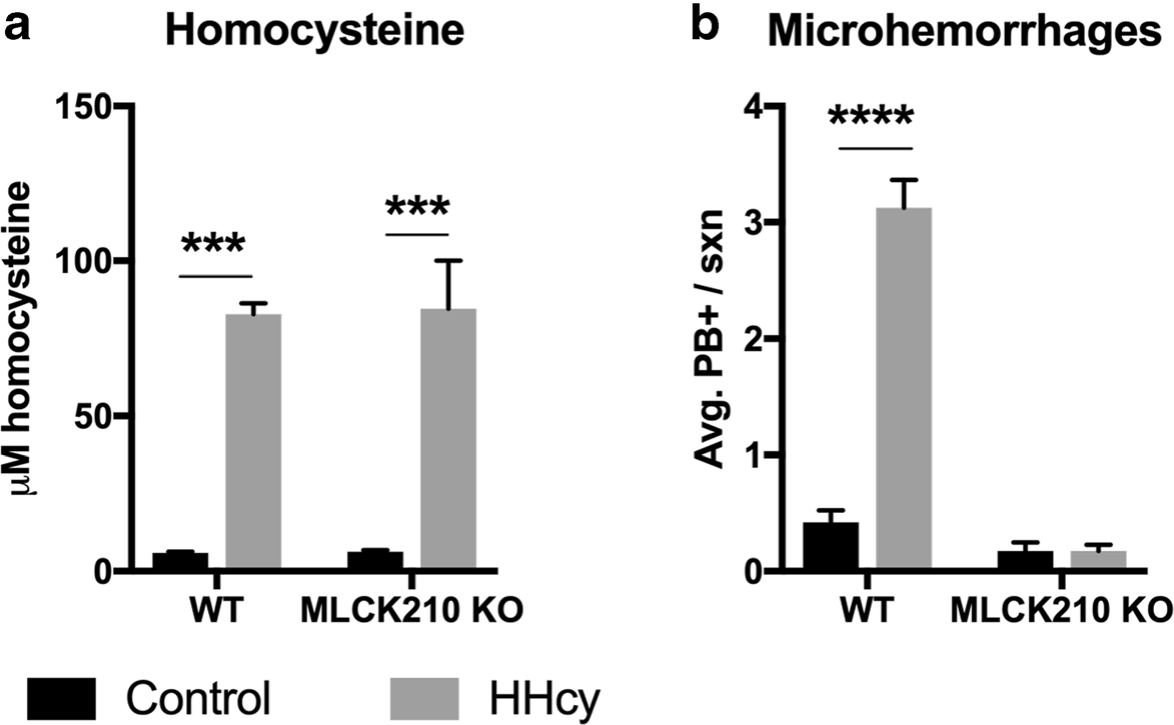
HHcy diet induces hyperhomocysteinemia in both strains but MLCK210 KO mice are protected from microhemorrhages. **a** Plasma homocysteine was measured in a subset of mice (*n* = 3) from each group. After 6 weeks of HHcy diet, plasma homocysteine was significantly elevated in both strains relative to control diet. There were no differences between WT and MLCK210 KO mice at baseline. **b** Average Prussian blue positive microhemorrhages per section were elevated in WT mice in response to HHcy diet, but unchanged in MLCK210 KO mice. ***p < .001, ****p < .0001 vs. within-strain control, two-way ANOVA with Sidak’s post hoc tests

Plasma homocysteine concentration was measured in a subset of 3 mice per group, and levels on control diet were not significantly different between WT (M = 5.89 μM, SD = 0.67, CI = 4.23–7.55) and MLCK210 KO mice (M = 6.34 μM, SD = 0.74, CI = 4.50–8.18) (Fig. 1a). The HHcy diet significantly increased plasma homocysteine levels in both the WT (M = 82.93 μM, SD = 6.17, CI = 67.61–98.25) and KO mice (M = 84.67 μM, SD = 26.57, CI = 18.65–150.7), with no significant difference between the strains. The HHcy model has been reported to induce cerebrovascular pathology such as cerebral microhemorrhages (Sudduth et al. 2013). Therefore, we measured microhemorrhages by Prussian blue staining. The average number of Prussian blue positive profiles per section was significantly elevated in the WT mice on HHcy diet (M = 3.124, SD = 0.760, CI = 2.580–3.667) versus control diet (M = 0.421, SD = 0.297, CI = 0.173–0.669) (Fig. 1b). In contrast, the MLCK210 KO mice were completely protected from HHcy-induced microhemorrhages, with no difference found between those mice on HHcy diet (M = 0.175, SD = 0.155, CI = .045–0.305) or control diet (M = 0.175, SD = .208, CI = 0.001–0.349).

**Fig. 2:**
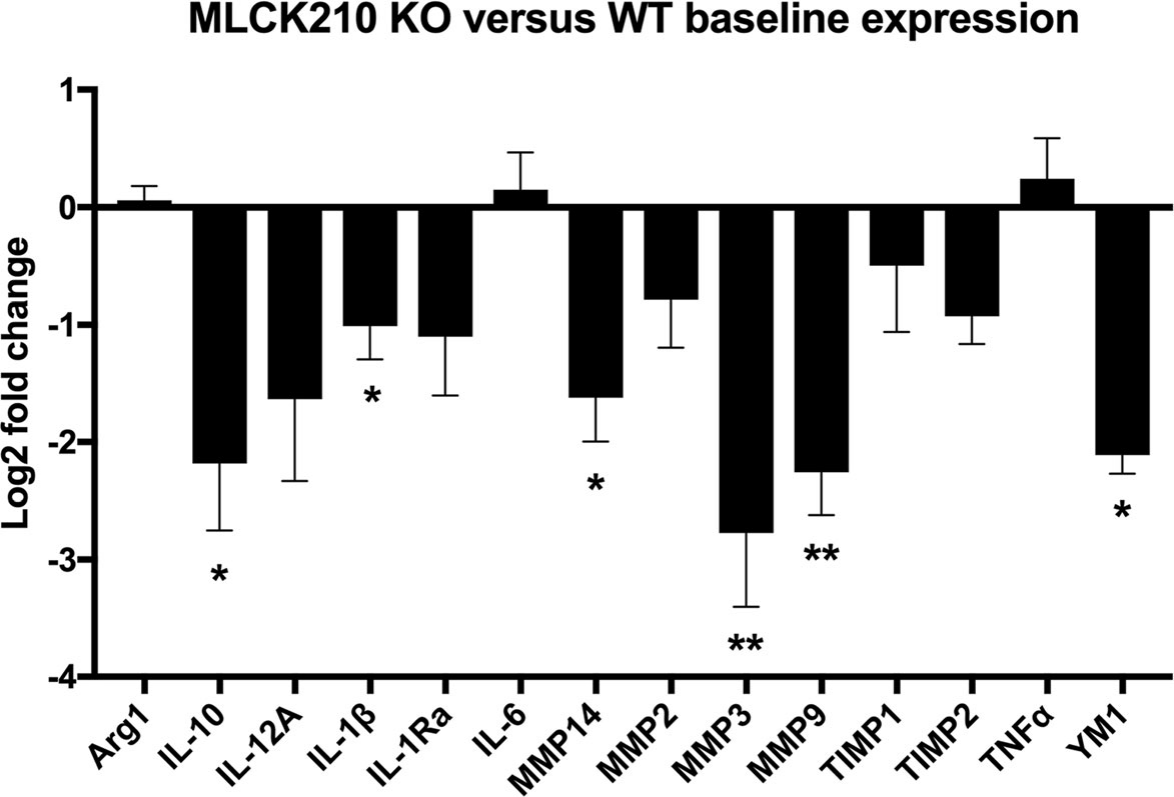
Differences in baseline gene expression levels between WT and MLCK210 KO mice on control diet. Cortical expression levels in MLCK210 KO mice relative to WT mice on control diet are shown. The basal mRNA levels of six genes (IL-10, IL-1β, MMP14, MMP3, MMP9, YM1) were significantly lower in MLCK210 KO mice compared to WT mice. *p < .05, **p < .01 vs. WT mice on control diet, multiple independent sample *t* tests, followed by two-stage step-up analyses (Benjamini et al. 2006) with *Q* = 10%

**Fig. 3:**
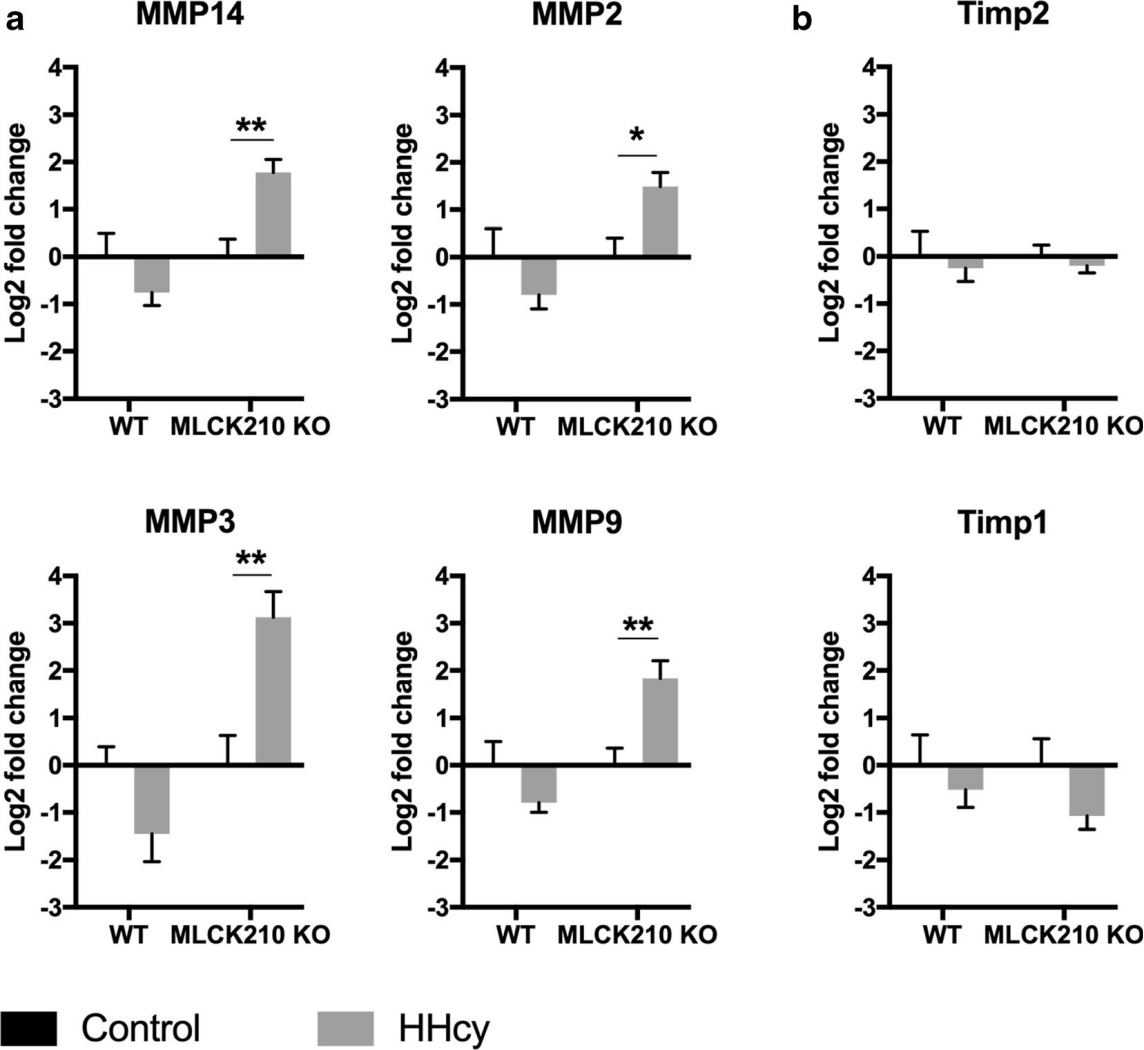
MMP transcript levels are unchanged in WT and upregulated in MLCK210 KO mice on HHcy diet, with no changes in TIMP transcript levels. **a** Cortical MMP expression levels show that in WT mice, there were no HHcy-induced increases in any of the MMP transcripts quantified. In MLCK210 KO mice, all of the MMPs were significantly upregulated as a result of HHcy diet. **b** Transcript levels of tissue inhibitor of metalloproteinases (TIMP)-1 and TIMP-2 were unchanged by HHcy in either strain. *p < .05, **p < .01 vs. within-strain control, two-way ANOVA with Sidak’s post hoc tests

A gene expression panel related to neuroinflammation and BBB disruption, previously shown to change in response to HHcy, was screened for differences between WT and MLCK210 KO mice. The panel contained the inflammation-associated genes Arg1, IL-1β, IL-10, IL-12A, IL-1Ra, IL-6, TNF-α, and YM1, and the hemorrhage-implicated matrix remodeling genes MMP2, MMP3, MMP9, MMP14, TIMP1, and TIMP2 (Sudduth et al. 2013; Weekman et al. 2017). We first compared gene expression levels between the two genotypes at baseline, with values of the MLCK210 KO mice on control diet normalized to WT mice on control diet (Fig. 2). Unexpectedly, 6 genes were differentially expressed between the strains on control diet at baseline: MMP14, MMP3, MMP9, IL-1β, IL-10, and YM1. The basal mRNA levels of these 6 genes were significantly lower in MLCK210 KO compared to WT mice. Because baseline levels were different between strains, we normalized expression levels from mice on HHcy diet to within-genotype controls for characterization of diet-associated changes. Interestingly, levels of MMP14, MMP2, MMP3, and MMP9 were all significantly upregulated in the MLCK210 KO mice on the HHcy diet, in contrast to the WT mice on HHcy diet that showed no changes in MMP levels (Fig. 3a). No HHcy-induced changes were detected in either the TIMP1 or TIMP2 mRNA levels (Fig. 3b).

### MLCK210 KO reduces HHcy-induced pro-inflammatory changes

**Fig. 4:**
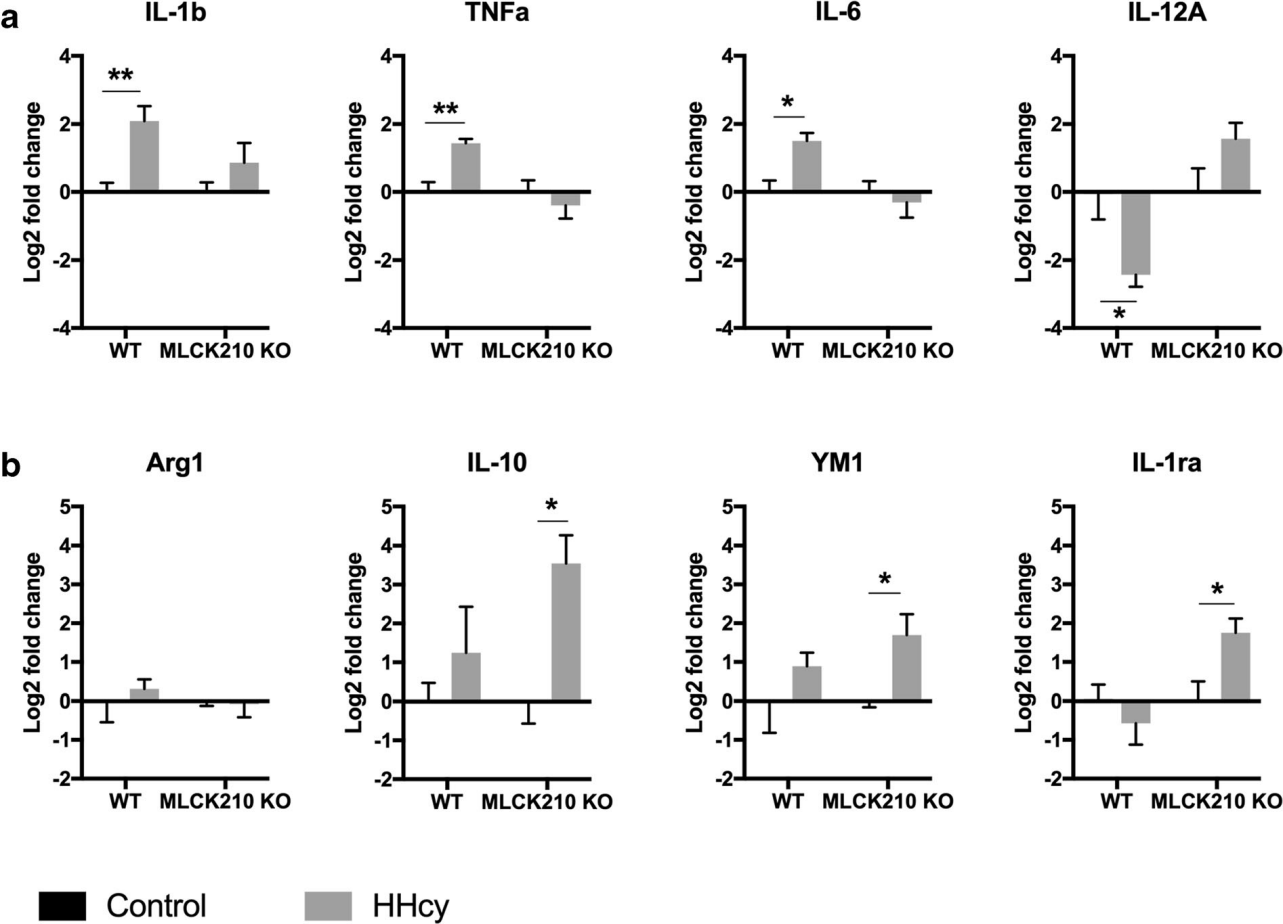
MLCK210 KO mice are protected from HHcy-induced neuroinflammation. **a** Cortical levels of pro-inflammatory cytokines IL-1β, TNF-α, and IL-6 were upregulated in WT mice on HHcy diet, and IL-12A was downregulated. There was no significant change in these cytokines in the MLCK210 KO mice on HHcy diet. **b** Anti-inflammatory mediators IL-10, IL-1Ra, and YM1 were significantly upregulated in the MLCK210 KO mice with HHcy, but not in the WT mice. Arg1 was unchanged with HHcy in either strain. *p < .05, **p < .01 vs. within-strain control, two-way ANOVA with Sidak’s post hoc tests

Increased levels of pro-inflammatory cytokines have been implicated as both a driver and consequence of BBB hyperpermeability, and MLCK210 specifically plays a role in BBB dysfunction induced by IL-1β (Beard et al. 2014) and pro-inflammatory processes mediated via NFκB signaling (Recoquillon et al. 2015). To determine whether the rescue of BBB integrity in this model corresponds with an overall shift from pro- to anti-neuroinflammatory responses, we measured mRNA levels of pro-inflammatory cytokines IL-1β, TNF-α, IL-6, and IL-12A, and the generally anti-inflammatory immune modulators Arg1, IL-10, IL-1Ra, and YM1. In line with previous studies (Sudduth et al. 2013; Sudduth et al. 2017), mRNA levels of IL-1β, TNF-α, and IL-6 were significantly upregulated in response to HHcy diet in the WT mice (Fig. 4a). This effect was reduced in the MLCK210 KO mice, in which no significant HHcy-induced changes in these cytokines were detected. Interestingly, we found IL-12A mRNA significantly downregulated in the WT mice after HHcy, an effect reversed in the MLCK210 KO mice (Fig. 4a). In line with the attenuated pro-inflammatory response to HHcy diet in the MLCK210 KO mice, there was a concomitant increase in the anti-inflammatory factors IL-10, IL-1Ra, and YM1, an effect not observed in WT mice (Fig. 4b). Arg1 levels were unchanged in either strain in response to HHcy diet (Fig. 4b).

## Discussion

Blood-brain barrier dysfunction is a critical factor in neurological dysfunction and neurodegeneration, contributing to multiple causes of cognitive impairment and dementia primarily affecting older populations. MLCK210 is an established regulator of tissue barrier permeability, the suppression of which is related to its potential as a therapeutic target in diverse preclinical models. The present study was designed to address whether such an approach might also be useful in some of the chronic stressors contributing to cerebrovascular damage. For this purpose, we subjected the MLCK210 KO mice to the dietary HHcy model of VCID that recapitulates many core pathophysiological features of cerebrovascular damage, including neuroinflammation and BBB disruption. We found that knockout of MLCK210 abrogated HHcy-induced microhemorrhage formation. In addition, MLCK210 KO mice showed a reduced neuroinflammatory profile after HHcy, as reflected in decreased transcript levels of pro-inflammatory cytokines (IL-1β, IL-6, TNF-α) and increased levels of anti-inflammatory modulators (IL-10, IL-1Ra, YM1). Analysis of baseline expression levels of these genes between WT and MLCK210 KO mice indicated that these results were not likely due to a generally immunosuppressed phenotype in the KO animals. Overall, our findings using the MLCK210 KO mice extend the preponderance of data across diverse tissue barrier injury models that there is a potential for therapeutic benefit by targeting MLCK210 in future drug discovery efforts.

There were two interesting observations during the course of this study that remain to be addressed in future investigations. First, the finding that MMP levels were unchanged in the WT mice on HHcy diet despite significant microhemorrhage pathology and, conversely, MMP levels were elevated in the MLCK210 KO mice on HHcy diet without significant microhemorrhages was unexpected. Caution needs to be taken in the interpretation, however, as mRNA levels of the MMPs do not necessarily reflect the levels of the protein or protein activity (for review see Rempe et al. 2016). Therefore, it is not known if MMP enzyme activity differs between WT and MLCK210 KO mice on HHcy diet. To address whether MLCK210 might play a role in the homeostatic maintenance of brain extracellular matrix as mediated via the MMP system, future studies should directly measure MMP enzyme activity at baseline and in response to HHcy in MLCK210 KO mice. Second, IL-12A was an exception to the trend where most of the immunomodulatory gene expressions were altered. We previously found an increase in IL-12A transcript levels in hippocampus after 6 weeks on HHcy diet (Sudduth et al. 2017) compared to the decrease in cortical levels observed here (Fig. 3d). The main difference might simply reflect the anatomical region of measurement. This raises the potential of regional variability in brain neuroinflammatory responses to HHcy. IL-12A is also interesting in that it encodes a protein that can function as part of two different heterodimer cytokines: the pro-inflammatory IL-12 and the anti-inflammatory IL-35 (for review see Vignali and Kuchroo 2012). While not widely studied in the brain, IL-35 has recently been shown to have neuroprotective effects in a cerebral ischemia mouse model (Xu et al. 2018). Therefore, whether this HHcy-induced cortical decrease in IL-12A in the WT mice is reflective of pro- or anti-inflammatory processes remains to be determined.

In summary, the present study demonstrates that suppression of MLCK210 can provide protection in a model of chronic cerebrovascular dysfunction, and supports the hypothesis that MLCK210 is a viable target for progressive CNS diseases such as VCID. Future studies should explore MLCK210 inhibition as a therapeutic strategy for chronic neurological diseases involving cerebrovascular pathology and BBB dysfunction mechanisms.
